# Overexpression of NTPCS1 Enhances Zn Tolerance in Tobacco

**DOI:** 10.3390/plants14111688

**Published:** 2025-05-31

**Authors:** Chanjuan Wu, Jie Zhang

**Affiliations:** College of Life Sciences and Agri-Forestry, Southwest University of Science and Technology, Mianyang 621010, China; zhangjie0930123@163.com

**Keywords:** phytochelatins, glutathione, phytochelatin synthase, Zn tolerance, *Nicotiana tabacum*

## Abstract

Phytochelatins (PCs) are well-characterized for their role in detoxifying non-essential metals like cadmium (Cd), but their role in zinc (Zn) homeostasis remains underexplored. In this study, we investigated the role of the *Nicotiana tabacum* phytochelatin synthase 1 (*NtPCS1*) in counteracting Zn toxicity in plants. qRT-PCR data showed that the transcript level of the *NtPCS1* gene was upregulated by ZnSO_4_, leading to increased PC production in the wild-type tobacco plants. Functional complementation assays in *Arabidopsis thaliana* revealed that overexpression of *NtPCS1* rescued the Zn hypersensitivity of the *Atpcs1* mutant, with the N-terminal region being indispensable for Zn tolerance. In addition, transgenic tobacco plants overexpressing *NtPCS1* (PCS1 lines) exhibited superior root elongation under ZnSO_4_ stress compared to the wild-type plants, particularly when supplemented with glutathione (GSH). The observed phenotypic advantage is attributed to *NtPCS1*-mediated overproduction of PCs, which facilitated Zn chelation and enabled cellular detoxification. These findings highlight the important role of NtPCS1 in Zn tolerance via GSH-linked PCs synthesis, offering insights into *PCS*-mediated Zn detoxification and a genetic strategy for developing Zn-resistant plants.

## 1. Introduction

Zinc (Zn), an essential micronutrient for plant growth and development, exhibits phytotoxicity at elevated concentrations, disrupting cellular homeostasis and impairing physiological processes [1]. Plants employ detoxification strategies, such as chelation and efflux mechanisms, to maintain metal ion homeostasis and mitigate metal(loid) toxicity [2,3]. Phytochelatins (PCs) are cysteine-rich peptides (general structure: (γ-Glu-Cys)*_n_*-Gly, with *n* = 2–11) that function as critical metal-binding agents in plants [3]. It chelates metal ions such as Zn via cysteine-containing sulfhydryl groups to form stable cytosolic complexes, which are subsequently transported into vacuoles. This sequestration and compartmentalization reduce the concentration of free Zn ions in the cytoplasm, protecting cellular components from Zn toxicity and contributing to plant cell detoxification [3,4]. PCs are biosynthesized from glutathione (GSH) by phytochelatin synthase (PCS) [3,4]. Genes encoding PCS have been found in diverse organisms ranging from bacteria [5] to fungi [6], algae [7,8], plants [9,10,11,12], and metazoans [13,14,15].

The expression of *PCS* genes exhibits species- and tissue-specific variations. In *Arabidopsis thaliana* [16], *Brassica juncea* [17], and *Tagetes patula* [18], root tissues show higher *PCS* expression compared to shoots, while *Helianthus annuus* displays the opposite pattern [19]. In *Oryza sativa*, there is significant variation in the expression levels of *OsPCS1* and *OsPCS2* across different organs [20,21]. In addition, the expression of *PCS* genes is regulated by different metal exposure. Non-essential heavy metals like Cd, As, Hg, and Pb strongly induce expression, such as *B*. *juncea BjPCS* [17,22], rice *OsPCS5/7/9/15* [11], *Paspalum vaginatum PvPCS1/2* [23], and *Saccharum officinarum SoPCS* [24]. Meanwhile, essential micronutrients such as Zn and Cu also moderately upregulate the expression of *PCS* genes, although not as strongly as heavy metals do. In *Solanum lycopersicum*, the expression of *SlPCS1* was strongly induced by Cd and Pb, and was also induced to a certain extent by Cu [25]. Similarly, *MaPCS* expression was induced by Zn and Cd stress, with a stronger response to Cd in *Morus alba* [26]. *OsPCS9* expression was stimulated by Cd and Zn in rice [11]. In *Azolla* species, *A. pinnata* showed high Cu uptake, and *A. filiculoides* and samples from the Anzali wetland showed high Zn uptake [27]. Other factors, including sulfur availability and microbial interactions, also modulate *PCS* gene expression [28], reflecting its responsiveness to diverse environmental stimuli.

Overexpression or heterologous introduction of *PCS* genes has been proposed as a promising strategy for enhancing metal tolerance and detoxification. Substantial evidence demonstrates the effectiveness of this approach for toxic metals like Cd and As. For instance, the expression of the Arabidopsis *AtPCS1* confers Cd tolerance and facilitate Cd detoxification in both *Escherichia coli* and *Saccharomyces cerevisiae* [29,30]. The expression of the *Brassica rapa BrPCS1* gene imparts yeast with resistance to Cd [10]. The Arabidopsis plants expressing *Boehmeria nivea BnPCS1* [31], *Brassica napus BnPCS* [32], and *Caenorhabditis elegans CePCS* [13], as well as *B. juncea* plants expressing *AtPCS1* [33] and *Nicotiana glauca* plants expressing wheat *TaPCS1* [34], have been observed to exhibit a marked increase in Cd and As tolerance. Meanwhile, evidence suggests that *PCS* genes also confer tolerance to essential micronutrients like Zn. Notably, heterologous expression of *BrPCS1* in yeast improves tolerance to Zn [10], and *AtPCS1* expression in *B. juncea* confers Zn tolerance [33]. Furthermore, overexpression of mulberry *MnPCS1/2* in Arabidopsis and tobacco enhances Zn tolerance [26]. These findings underscore the expansive potential of PCS-mediated metal tolerance strategies for mitigating the effects of environmental contamination.

Compared to the well-established role of PCs in Cd detoxification, Zn-specific responses remain largely unexplored. Although the tobacco *NtPCS1* gene is known to play a crucial role in plant development and Cd response [35,36], its role in Zn detoxification has yet to be elucidated. This study aimed to address this gap by systematically investigating Zn-induced expression of *NtPCS1* and PCs synthesis, evaluating Zn tolerance in transgenic Arabidopsis and tobacco plants overexpressing *NtPCS1*, identifying the critical structural domains of the NtPCS1 protein required for Zn tolerance, and examining the effect of GSH on Zn homeostasis in tobacco. Our findings demonstrate that NtPCS1 mediates Zn tolerance through GSH-dependent PC synthesis, with the interplay between GSH availability and PC production emerging as a critical factor in mitigating Zn toxicity. This finding reveals a molecular mechanism underlying Zn homeostasis that is different from those previously reported. Specifically, the N-terminal region of NtPCS1 was identified as essential for Zn tolerance, underscoring the structural specificity of PCS activity in Zn homeostasis. These insights advance our understanding of PCS-mediated Zn homeostasis, providing a foundation for developing genetic strategies to enhance plant resilience in metal-contaminated environments.

## 2. Results

### 2.1. Zn Induces an Increase in NtPCS1 Expression and PCs Synthesis

**Figure 1:**
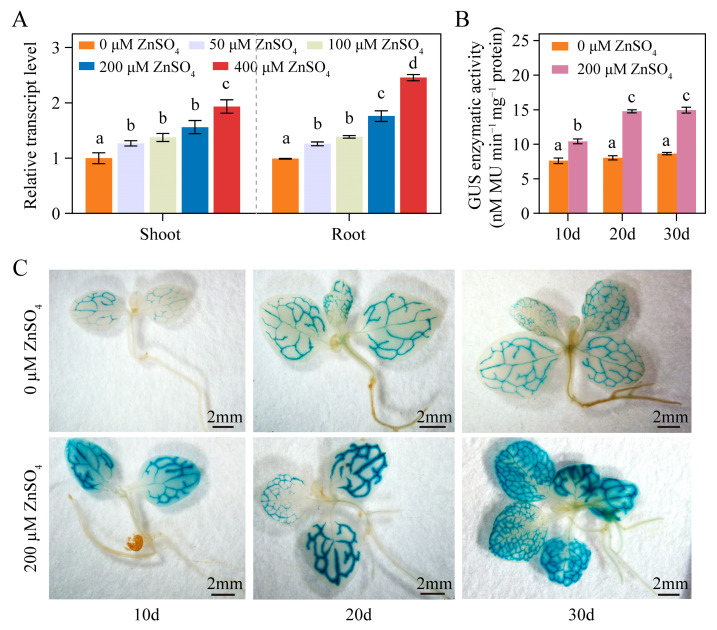
The Zninduced expression of the *NtPCS1* gene. (**A**) The effect of ZnSO_4_ treatment on the transcript level of *NtPCS1* in WT tobacco plants was detected by qRT-PCR. WT plants were treated with various concentrations of ZnSO_4_ (0, 50, 100, 200, and 400 μM) for 48 h. Values represent means ± SD of three biological replicates. Different letters indicate statistically significant differences (one-way ANOVA followed by a Tukey’s HSD test, *p* < 0.05). Separate statistical analyses were conducted for shoot and root samples. (**B**,**C**) The effect of ZnSO_4_ treatment on the transcription activation of the *GUS* gene driven by the 1097 bp promoter of *NtPCS1* was detected via GUS enzymatic activity (**B**) and GUS staining (**C**) assays. Transgenic tobacco seeds expressing pBI121-*NtPCS1pro::GUS* were germinated and grown on 1/2 MS medium supplemented with 0 or 200 μM ZnSO_4_ for 10, 20, or 30 days. Values represent means ± SD of three biological replicates. Different letters indicate statistically significant differences (two-way ANOVA followed by a Tukey’s HSD test, *p* < 0.05). Three independent transgenic tobacco lines expressing pBI121-*NtPCS1pro::GUS* were used in (**B**,**C**), and similar results were obtained.

In a previous study, we found that the enzymatic activity of the NtPCS1 protein was significantly enhanced by the metal ion Zn^2+^ [36]. To explore the impact of Zn on the expression of the *NtPCS1* gene in tobacco, WT plants were treated with various concentrations of ZnSO_4_ (0, 50, 100, 200, and 400 μM) for 48 h. Subsequently, quantitative real-time PCR (qRT-PCR) was conducted to detect the transcript level of *NtPCS1*. The results revealed that the transcript level of *NtPCS1* was upregulated by ZnSO_4_ in tobacco (Figure 1A). When the plants were treated with 50, 100, 200, and 400 μM ZnSO_4_, the transcript level of *NtPCS1* increased by approximately 1.26, 1.37, 1.56, and 1.93-fold in shoots and 1.27, 1.39, 1.78, and 2.48-fold in roots, respectively, relative to untreated control (Figure 1A). These findings suggest that the expression of *NtPCS1* is regulated by ZnSO_4_ stress.

Furthermore, to investigate the Zn-responsive transcriptional regulation of *NtPCS1*, transgenic tobacco seedlings harboring the pBI121-*NtPCS1pro:GUS* construct were cultivated on 1/2 MS medium supplemented with 0 or 200 μM ZnSO_4_. Seedlings harvested at 10-, 20-, and 30-day intervals exhibited a time-dependent increase in GUS activity, as determined by enzymatic assays and histochemical staining (Figure 1B,C). In Zn-free control conditions, GUS activity showed a gradual but non-significant increase across the three time points (Figure 1B). In contrast, ZnSO_4_-exposed seedlings exhibited substantially elevated GUS activity at all detected time points compared to controls, which showed a significant increase from day 10 to day 20. A slight increase in GUS activity was also observed from day 20 to day 30, though statistically insignificant (Figure 1B). Histochemical staining patterns corroborated the enzymatic activity data, displaying intensified GUS signals in Zn-treated seedlings throughout the experimental period (Figure 1C). These findings confirm ZnSO_4_-mediated transcriptional activation of the *NtPCS1* gene.

**Figure 2:**
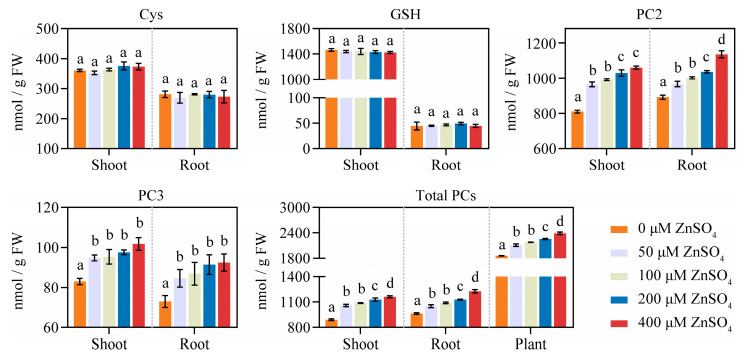
PC content of the WT tobacco plants under ZnSO_4_ stress. The effect of ZnSO_4_ treatment on the synthesis of PCs in WT tobacco plants was analyzed by HPLC. WT tobacco plants were treated with various concentrations of ZnSO_4_ (0, 50, 100, 200, and 400 μM) for 48 h. Total PCs is equal to the sum of PC2 and PC3. Values represent means ± SD of three biological replicates. Different letters indicate statistically significant differences (one-way ANOVA followed by a Tukey’s HSD test, *p* < 0.05). Separate statistical analyses were conducted for shoot, root, and plant samples.

To determine whether the Zn-induced upregulation of *NtPCS1* facilitates the biosynthesis of PCs, we treated WT tobacco plants with various concentrations of ZnSO_4_ (0, 50, 100, 200, and 400 μM) for 48 h, and conducted a quantitative analysis of the PC content using high performance liquid chromatography (HPLC). When plants were grown under ZnSO_4_ stress, although the contents of Cys and GSH in shoots and roots remained unchanged, both shoots and roots contained significantly higher levels of PC2, PC3, and total PCs compared with the ZnSO_4_-free control (Figure 2). This finding demonstrates that ZnSO_4_ stress enhances PC production in WT tobacco plants through upregulation of *NtPCS1* expression.

### 2.2. Overexpression of NtPCS1 Enhances Zn Tolerance in Tobacco

The upregulation of *NtPCS1* expression and the increased PC production under ZnSO_4_ stress promoted us to investigate the effect of *NtPCS1* overexpression on Zn tolerance in tobacco. For this purpose, we used three *NtPCS1*-overexpressing lines from our latest research: PCS1-1, PCS1-8, and PCS1-11, which have been designated as the PCS lines [36]. Seedlings of WT and PCS1 lines were grown on 1/2 MS medium supplemented with 0 or 200 μM ZnSO_4_. As results, all seedlings exhibited growth inhibition under the 200 μM ZnSO_4_ treatment, manifesting as reduced root lengths, when compared to the untreated control (Figure S1A,B). Meanwhile, there were no significant differences in growth between WT and PCS1 lines when seedlings were grown on media containing either 0 or 200 μM ZnSO_4_ (Figure S1A,B).

**Figure 3:**
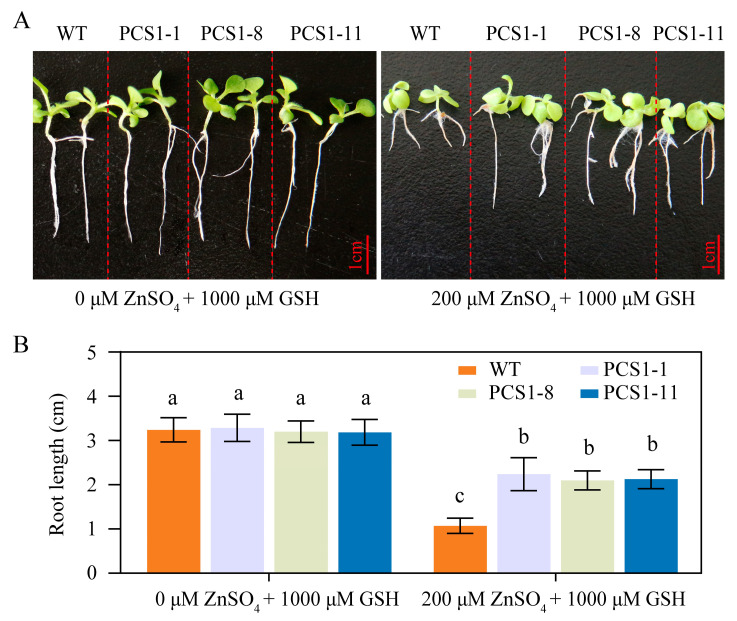
Phenotypes of PCS1 lines under ZnSO_4_ stress in the presence of GSH. (**A**,**B**) Phenotypes (**A**) and root length (**B**) of WT and PCS1 lines under ZnSO_4_ stress in the presence of GSH. Tobacco seeds from WT and PCS1 lines were germinated and grown on 1/2 MS medium, either in the absence or presence of 200 μM ZnSO_4_, supplemented with 1000 μM GSH, for a period of 21 days. The PCS1 lines refers to the transgenic tobacco transformed with the pBI121-*35Spro::NtPCS1* construct. Values represent means ± SD (*n* = 24). Different letters indicate statistically significant differences (two-way ANOVA followed by a Tukey’s HSD test, *p* < 0.05).

Given that *AtPCS1*-overexpressing tobacco plants demonstrated heightened sensitivity to Cd compared to control [37], yet showed enhanced Cd tolerance with the addition of exogenous GSH to the growth medium [38], the impact of GSH on the response to Zn stress was investigated in this study. Upon supplementation with 1000 μM GSH, the growth of both WT and PCS1 lines was comparable under 0 μM ZnSO_4_ (Figure 3A,B). Notably, under 200 µM ZnSO_4_, the PCS1 lines exhibited a significant improvement in root growth compared to the WT (Figure 3A,B). This observation suggests that the overexpression of *NtPCS1* can enhance Zn tolerance in tobacco plants in the presence of GSH.

### 2.3. The N-Terminal Domain of the Tobacco NtPCS1 Protein Is Crucial for Zn Tolerance

The NtPCS1 protein possesses two characteristic domains: the N-terminal Pfam 05023 (phytochelatin) and the C-terminal Pfam 09328 (phytochelatin_C) [36]. To identify the critical domain of NtPCS1 that is essential for Zn tolerance, we generated three transgenic Arabidopsis materials, which separately express the full-length *NtPCS1* (*NtPCS1-F*, 1-501 aa), the N-terminal truncation (*NtPCS1-N*, 1-219 aa), and the C-terminal truncation (*NtPCS1-C*, 220-501 aa), each driven by the CaMV 35S promoter, in the *Atpcs1* mutant background. These materials were sourced from our recently published research [36].

**Figure 4:**
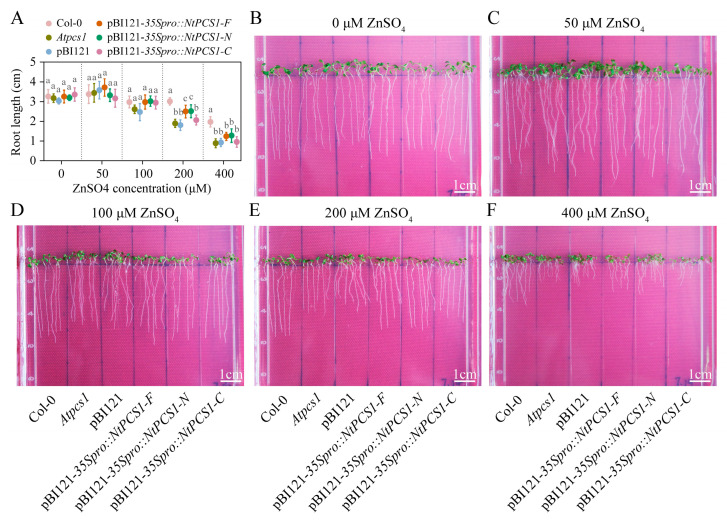
Functional complementation of the Arabidopsis *Atpcs1* mutant by heterologous overexpression of *NtPCS1*. (**A**–**F**) Root length (**A**) and phenotypes (**B**–**F**) of WT (Col-0), *Atpcs1* mutant, and transgenic Arabidopsis seedlings expressing pBI121-*35Spro::NtPCS1-F/N/C* under ZnSO_4_ stress. Seeds were germinated and grown on 1/2 MS medium supplemented with different concentrations of ZnSO_4_, encompassing: 0 (**B**), 50 (**C**), 100 (**D**), 200 (**E**), and 400 (**F**) μM ZnSO_4_. After 9 days, the root length of seedlings was measured. NtPCS1-F represents the full length of NtPCS1 (1-501 aa), NtPCS1-N represents the N-terminal region of NtPCS1 (1-219 aa), and NtPCS1-C represents the C-terminal region of NtPCS1 (220-501 aa). Col-0: Columbia-0; *Atpcs1*: *Atpcs1* mutant; pBI121: *Atpcs1* mutant transformed with empty pBI121 vector; pBI121-*35Spro::NtPCS1-F/N/C*: *Atpcs1* mutant transformed with pBI121-*35Spro::NtPCS1-F/N/C*. Values represent means ± SD (*n* = 24). Different letters indicate statistically significant differences (one-way ANOVA followed by a Tukey’s HSD test, *p* < 0.05).

Seedlings of WT (Col-0), *Atpcs1* mutant, and transgenic Arabidopsis seedlings were vertically cultured on 1/2 MS media supplemented with 0, 50, 100, 200, and 400 μM ZnSO_4_, respectively. Under a 50 μM ZnSO_4_ condition, all seedlings exhibited enhanced root elongation compared to the Zn-free control, likely due to optimized Zn availability for growth (Figure 4A–C). When grown in medium containing 100, 200, or 400 μM ZnSO_4_, all seedlings exhibited growth inhibition compared to the Zn-free control, manifesting as reduced root lengths, with the inhibitory effect being dose-dependent (Figure 4A,D–F). In addition, when seedlings were grown under 0, 50, and 100 μM ZnSO_4_, no significant difference was observed among WT, *Atpcs1* mutant, and transgenic seedlings (Figure 4A–D). Notably, under 200 and 400 μM ZnSO_4_, the *Atpcs1* mutant exhibited obviously shorter root length than WT, indicating the hypersensitivity to ZnSO_4_ stress (Figure 4A,E,F). Interestingly, under 200 μM ZnSO_4_, transgenic Arabidopsis seedlings expressing pBI121-*35Spro::NtPCS1-F* and pBI121-*35Spro::NtPCS1-N* displayed intermediate root lengths between the *Atpcs1* mutant and WT (Figure 4A,E). Similarly, the root lengths of these transgenic seedlings also fell between those of the *Atpcs1* mutant and the WT when grown in medium containing 400 μM ZnSO_4_, although the difference was not statistically significant (Figure 4A,F). These findings suggest that the heterologous overexpression of *NtPCS1-F* and *NtPCS1-N* can partially rescue the Zn-hypersensitive phenotype of the *Atpcs1* mutant. However, neither pBI121-*35Spro::NtPCS1-C* nor the empty pBI121 vector transformation complemented the Zn-hypersensitive phenotype of the *Atpcs1* mutant (Figure 4A,E,F). These results demonstrate that heterologous overexpression of tobacco *NtPCS1* can enhance Zn tolerance in Arabidopsis, and the N-terminal domain of the NtPCS1 protein is crucial for mediating Zn tolerance in plants.

### 2.4. Overexpression of NtPCS1 Results in Increased PC and Zn Content in Tobacco

To investigate whether the enhanced Zn tolerance in PCS1-line tobacco plants in the presence of GSH is attributed to the increased PC synthesis and Zn detoxification, WT and PCS1-line tobacco plants were exposed to hydroponic solutions containing either 0 or 200 μM ZnSO_4_, with the addition of 1000 μM GSH. After 14 days of treatment under these conditions, the contents of PCs and Zn were assessed.

**Figure 5:**
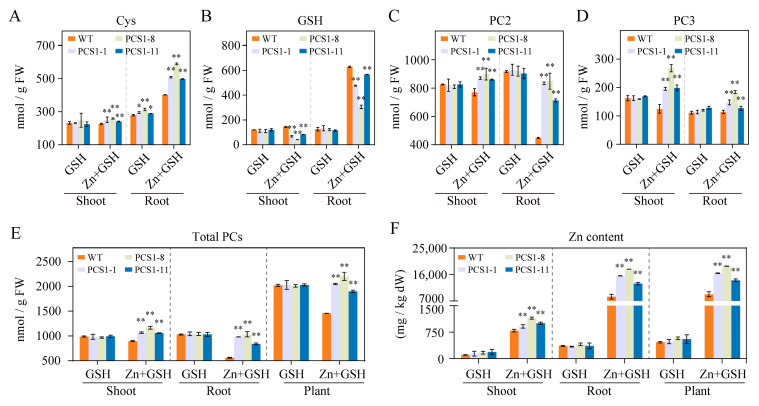
PC and Zn content of WT and PCS1 lines under ZnSO_4_ stress in the presence of GSH. (**A**–**E**) The content of Cys (**A**), GSH (**B**), PC2 (**C**), PC3 (**D**), and total PCs (**E**) in WT and PCS1 lines were analyzed by HPLC. Total PCs is equal to the sum of PC2 and PC3. (**F**) The content of Zn in WT and PCS1 lines was analyzed by AAS. All the Zn content data presented in the figure have been normalized based on the dry weight of the samples to ensure accurate and comparable measurements. Tobacco plants were treated with 0 or 200 μM ZnSO_4_ in the presence of 1000 µM GSH for 14 days. The PCS1 lines refer to the transgenic tobacco transformed with the pBI121-*35Spro::NtPCS1* construct. GSH: 0 μM ZnSO_4_ + 1000 µM GSH; Zn + GSH: 200 μM ZnSO_4_ + 1000 µM GSH. Values represent means ± SD of three biological replicates. *, *p* < 0.05, and **, *p* < 0.01, indicate statistically significant differences between PCS1-line and WT tobacco plants grown under the same conditions.

In the presence of GSH alone, the Cys content exhibited tissue-specific variation between the WT and PCS1 lines, with the PCS1 lines exhibiting comparable levels in shoot tissues but a significant elevation in root tissues compared to WT (Figure 5A). Both WT and PCS lines maintained comparable concentrations of GSH, PC2, PC3, and total PCs within both shoot and root tissues (Figure 5B–E). When subjected to ZnSO_4_ stress, the PCS1 lines showed elevated Cys levels, whereas the levels of GSH were reduced in both shoots and roots compared to the WT (Figure 5A,B). Moreover, the PCS1 lines displayed a significant increase in PC2, PC3, and total PC contents in both shoots and roots (Figure 5C–E). These observed metabolic changes indicate that the reduced GSH is being utilized for the production of PCs.

The Zn content in both WT and PCS1 lines was assessed using absorption spectroscopy analysis (AAS). In the presence of GSH alone, the content of Zn in both WT and PCS1 lines was minimal, with no significant difference detected between them (Figure 5F). However, when exposed to ZnSO_4_ stress in combination with GSH, there was a marked increase in Zn content in both the shoots and roots of WT and PCS1 lines, as compared to the condition with GSH alone (Figure 5F). The roots contained higher concentrations of Zn compared to the shoots under these conditions (Figure 5F). Notably, the PCS1 lines exhibited a significantly greater Zn content in both shoots and roots compared to the WT when subjected to the ZnSO_4_ and exogenous GSH (Figure 5F). These findings suggest that the increase of PCs synthesis, which leads to enhanced Zn sequestration, plays a crucial role in the improved Zn tolerance of PCS1-line tobacco plants.

## 3. Discussion

PCS functions as an enzyme that catalyzes the biosynthesis of PCs to chelate metal ions, thereby playing a pivotal role in metal detoxification and homeostasis maintenance [3]. It exhibits broad phylogenetic conservation spanning bacteria, fungi, algae, plants, and metazoans [5,6,7,8,9,10,11,12,13,14,15], reflecting their fundamental biological significance across the tree of life. While PCS-mediated tolerance to non-essential metals like Cd, Pb, and As is well-documented, its role in managing essential metals like Zn remains poorly understood. We aim to investigate the function of the tobacco NtPCS1 in Zn homeostasis and detoxification.

The PCS proteins are characterized by a conserved N-terminal catalytic domain, known as phytochelatin, and a variable C-terminal domain, referred to as phytochelatin_C, which is rich in cysteine residues [3,36,39]. Studies indicated that the C-terminal domain of the Arabidopsis AtPCS1 plays a pivotal role in its activation by various heavy metal ions such as Cd, Hg, Zn, and As (III) [40,41,42,43]. For example, truncation of the C-terminus of AtPCS1 abolished Zn-induced synthesis of PC2 and PC3, while activation by Cd was unaffected [40]. In Arabidopsis, the *cad1-6* mutant, harboring a truncation in the C-terminal domain of AtPCS1, exhibited increased sensitivity to As(III), with minimal PC synthesis, suggesting that the *cad1-6* mutant is defective in its catalytic activity under As(III) stress [43]. In addition, the mutation of highly conserved cysteine residues in the C-terminus of AtPCS1 has uncovered that two twin-cysteine motifs differentially repress enzyme activation in response to heavy metal exposure [44]. This finding emphasizes the role of the C-terminus as a regulatory domain that represses enzyme overactivation by both essential and non-essential heavy metals, thereby highlighting its function as a crucial inhibitor of enzymatic overactivation [44]. In this work, functional complementation analysis showed that the full-length NtPCS1 and its N-terminal truncation could partially rescue the Zn-hypersensitive phenotype of the Arabidopsis *Atpcs1* mutant, but the C-terminal truncation was unable to do so, indicating that the N-terminal domain of NtPCS1 is essential for Zn tolerance. These findings together suggest that both the C-terminal and N-terminal domains of PCS proteins are important for their function, with their significance varying across different species, highlighting the complex interplay between these domains in conferring tolerance to metals such as Zn. Therefore, further exploration is required to elucidate the structure and function of the PCS protein regions in different biological contexts.

It is widely recognized that PCs are synthesized from GSH by the enzyme PCS [3,4]. When plants grown on metal stress, a common occurrence is the depletion of GSH, which often restricts PC production. In this study, we found that overexpression of *NtPCS1* in tobacco plants only enhanced Zn tolerance when supplemented with exogenous GSH, thereby emphasizing the substrate-dependent limitation on the biosynthesis of PCs. This finding aligns with observations from a previous study, which highlighted that the availability of GSH is a pivotal factor influencing the efficiency of PC production [38]. Notably, our study also reveals that the transgenic tobacco lines overexpressing *NtPCS1* did not suffer from any fitness costs under low-Zn or GSH-limited conditions, indicating that the overexpression of *NtPCS1* does not inherently impair plant growth. This is a vital consideration for agricultural applications. However, we also noted that Zn stress induced significant GSH depletion in these transgenic lines, which could lead to concerns about vulnerability to oxidative stress. This is due to the dual role of GSH in both PC synthesis and in redox buffering. This duality highlights the necessity for a delicate balance between PCS activity and GSH metabolism to avoid unintended metabolic trade-offs. Although *PCS* overexpression can improve metal tolerance, it requires a substantial amount of intracellular GSH for PC production. A decline in GSH levels may potentially exacerbate oxidative stress. Furthermore, prolonged *PCS* overexpression may disrupt metabolic pathways, altering resource allocation and impacting agronomic traits such as growth rate, biomass, and seed yield. Therefore, when applying *PCS* overexpression in the field, it is essential to comprehensively evaluate the balance between the enhanced metal tolerance and the potential negative effects to ensure that the overall adaptability of plants and agricultural production are not overly compromised. In conclusion, this work not only profiles the Zn-induced expression of *NtPCS1* but also reveals the important biological function of NtPCS1 in the response to Zn stress. It elucidates an interplay between GSH availability and Zn-specific detoxification mechanism. The study enhances our understanding of the molecular function of NtPCS1 and proposes the possibility of improving metal tolerance by overexpressing *PCS* genes in various species.

## 4. Materials and Methods

### 4.1. Plant Materials and Growth Conditions

In this study, the *N. tabacum* seeds utilized are of the NC89 ecotype, and the *A. thaliana* seeds employed belong to the Columbia-0 (Col-0) ecotype. The tobacco plants were cultivated in the greenhouse at 25 °C under a 16 h light/8 h dark photoperiod, while Arabidopsis plants were grown at 22 °C with the same light–dark cycle.

### 4.2. Plant Expression Constructs and Transgenic Plants

The transgenic tobacco plants used in this study were sourced from our recently published research [36]. In brief, the 1097 bp sequence fragment upstream of the ATG start codon of the *NtPCS1* gene was cloned into the pBI121 vector to drive the β-glucuronidase (*GUS*) reporter gene (pBI121-*NtPCS1pro::GUS*). In addition, the full-length coding sequence (CDS) of the *NtPCS1* gene were cloned into the pBI121 vector under the control of the CaMV35S promoter to generate the recombinant construct pBI121-*35Spro::NtPCS1*. Three independent overexpression lines of transgenic tobacco (PCS1 lines: PCS1-1, PCS1-8, and PCS1-11) were selected for subsequent experiments. Primers used for constructs are listed in Table S1.

### 4.3. Tobacco Seedling Experiments

All tobacco seedling experiments were performed independently three times and described as follows:

(i) To analyze the effect of Zn treatment on the expression of *NtPCS1* and PC content in tobacco, the WT tobacco seeds were germinated on a foam sheet. After 28 days, seedlings were transferred to a hydroponic system: roots were submerged in 1/2 Hoagland solution with renewal every 2–3 days. Following a 7-day acclimatization period, plants were transferred to renewed Hoagland solution containing ZnSO_4_ at final concentrations of 0, 50, 100, 200, or 400 µM. After 48 h, 100 mg samples of shoots and roots were collected for qRT-PCR to detect the transcript level of *NtPCS1*. Additionally, 1 g samples of shoots and roots were used for the detection of PCs content by high-performance liquid chromatography (HPLC).
(ii) To investigate the effect of Zn treatment on the activity of the *NtPCS1* promoter in tobacco, homozygous transgenic tobacco seeds, which were transformed with the pBI121-*NtPCS1pro::GUS* construct, were germinated on 1/2 MS medium supplemented with either 0 or 200 µM ZnSO_4_. After 10, 20, and 30 days of cultivation, 10 seedlings were harvested for GUS staining, and 1 g of seedlings was collected for enzymatic activity analysis.
(iii) In the root length experiment, seeds from both WT and homozygous PCS1 lines were germinated on 1/2 MS medium. The medium was supplemented with 0 or 200 μM ZnSO_4_, either with or without the addition of 1000 μM GSH. The seedlings were grown in a vertical orientation. After 21 days, the root lengths of the 24 tobacco seedlings were measured.
(iv) Seeds from both WT and homozygous PCS1 lines were germinated on a foam sheet. After 28 days, the seedlings were transferred to 1/2 Hoagland solution with renewal every 2–3 days. Following a 7-day acclimatization period, the tobacco seedlings were moved to a fresh solution containing either 0 or 200 µM ZnSO_4_, with or without the presence of 1000 µM GSH. After 14 days, 1 g and 5 g samples of both shoots and roots were collected for HPLC and absorption spectroscopy analysis (AAS) analyses, respectively, to quantify PCs and Zn contents.

### 4.4. Arabidopsis Atpcs1 Mutant Complementation

Four transgenic Arabidopsis materials in the *Atpcs1* mutant background (SAIL_650_C12), transformed with one of four constructs (pBI121, pBI121-*35Spro::NtPCS1-F*, pBI121-*35Spro::NtPCS1-N*, and pBI121-*35Spro::NtPCS1-C*), were used in this study. The Col-0 served as the control. Seeds of Col-0 and homozygous transgenic lines were germinated on 1/2 MS medium supplemented with 0, 50, 100, 200, or 400 µM ZnSO_4_ and grown vertically. The root length of 24 seedlings was measured after 9 days.

### 4.5. RNA Extract and qRT-PCR

Total RNA was isolated from WT and transgenic tobaccos using TRIzol reagent (Invitrogen, Waltham, MA, USA). Reverse transcription was performed with 2 µg RNA and 5X All-In-One Master Mix (G492, Abmart, Shanghai, China) to obtain cDNA. The qRT-PCR assay was conducted to analyze the transcript level of *NtPCS1* using KAPA SYBR FAST qPCR Master Mix (KK4601, KAPA Biosystems, Wilmington, MA, USA) on a CFX96 Real-Time PCR system (Bio-Rad, Hercules, CA, USA). It was performed independently three times. The *NtRL2* gene (Ribosomic protein L2, GenBank Z14081) was used as a control. Primers used for qRT-PCR are listed in Table S1.

### 4.6. GUS Staining

The collected samples were incubated in the staining solution, which contains 1 mM 5-bromo-4-chloro-3-indolyl glucuronide (X-gluc), 0.5 mM K_3_[Fe(CN)_6_], 0.5 mM K_4_[Fe(CN)_6_], 50 mM NaH_2_PO_4_ at pH 7.0, 50 mM Na_2_HPO_4_ at pH 7.0, 10 mM EDTA at pH 8.0, 0.1% (*v*/*v*) Triton X-100, and 20% (*v*/*v*) methanol. Subsequently, samples were incubated in solution with increasing ethanol concentrations (50%, 70%, 90%, and 100%) for chlorophyll decolorization.

### 4.7. GUS Enzymatic Activity Analysis

Tissue samples were homogenized in liquid nitrogen and suspended in GUS extraction buffer (0.1 M phosphate buffer pH 7.0, 10% (*w*/*v*) SDS, 0.5 M EDTA at pH 8.0, Triton X-100, β-mercaptoethanol). After centrifugation, the soluble protein fraction was collected and incubated with the GUS substrate for 60 min. Following the incubation, a luminescence spectrometer (LS-55, PerkinElmer, Waltham, MA, USA) was employed to conduct a quantitative fluorometric analysis aimed at investigating the effect of Zn treatment on the activity of the *NtPCS1* promoter in tobacco. This analysis was independently replicated three times. For the measurement of the total protein content, a dye-binding assay was utilized, with bovine serum albumin (BSA) serving as the standard.

### 4.8. PCs Content Analysis

A 100 mg collected sample was derivatized with monobromobimane (mBBr) and used for Cys, GSH, PC2, and PC3 analysis by HPLC as previously described [45]. This analysis was independently replicated three times.

### 4.9. Zn Content Analysis

Collected samples were oven-dried at 75 °C until reaching constant weight and ground to powder. A quantity of 100 mg dried powder was digested in HNO_3_ at 220 °C for 2 h. Samples were diluted with deionized water, and then Zn concentrations were measured by AAS (AA 700, PerkinElmer) analysis. This analysis was independently replicated three times. The Zn concentration data obtained from AAS analysis were normalized by the dry weight of the samples to ensure the comparability of the measurement results among different samples.

## Data Availability

The original contributions presented in the study are included in the article/Supplementary Material. Further inquiries can be directed to the corresponding author.

## Supplementary Materials

The following supporting information can be downloaded at: https://www.mdpi.com/article/10.3390/plants14111688/s1, Figure S1: Phenotypes of PCS1 lines under ZnSO_4_ stress; Table S1: List of primers used in this study.
