# Supplementary material for: Overexpression of NTPCS1 Enhances Zn Tolerance in Tobacco
Source: Plants (Basel). 2025 May 31;14(11):1688. doi: 10.3390/plants14111688 (PMC12157960; doi:10.3390/plants14111688)
Supplement: Supplementary file 1 [file plants-14-01688-s001.zip › plants-3673570-supplementary.pdf]

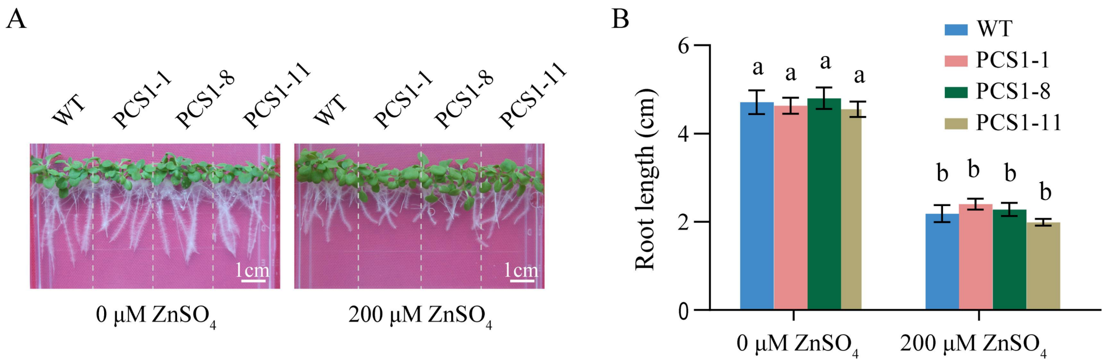

Figure S1. Phenotypes of PCS1 lines under $ZnSO_4$ stress. (**A, B**) Phenotypes (**A**) and root lengths (**B**) of WT and PCS1 lines under $ZnSO_4$ stress. Tobacco seeds from WT and PCS1 lines were germinated and grown on 1/2 MS medium, either in the absence or presence of 200 μM $ZnSO_4$, for a period of 21 days. Values represent means ± SD ($n = 24$). Different letters indicate statistically significant differences (two-way ANOVA followed by a Tukey's HSD test, $P < 0.05$).

Table S1. List of primers used in this study.

Primer name	Primer sequence (5'-3')	Construct	Application
NtPCS1-promoter-F	AAGCTTGTGCAGCAGCTGTTGAAGAAAG	pBI121-*NtPCS1pro::GUS*	Primers for plant constructs
NtPCS1-promoter-R	GGATCCTTTTTCTCGCTTCAGAATCTCC	pBI121-*NtPCS1pro::GUS*	
NtPCS1-CDS-F	GGATCCATGGCGATGGCGGGTTTA	pBI121-*35Spro::NtPCS1*	
NtPCS1-CSD-R	GTCGACCTAGAAGGGAGGTGCAGCTAAA	pBI121-*35Spro::NtPCS1*	
NtPCS1-RT-F	TGGTCTTGAATGCCCTTGC		Primers for real time PCR
NtPCS1-RT-R	GAGGCTCACAACAGTCCAACA		
NtRL2-RT-F	GTAAGGGAGCGGGTTCAGTCT		
NtRL2-RT-R	AACGGAGCACCCCTACCTG		
